# Comparison of Nucleosome, Ferritin and LDH Levels in Blood with Clinical Response before and after Electrochemotherapy Combined with IL-12 Gene Electrotransfer for the Treatment of Mast Cell Tumours in Dogs

**DOI:** 10.3390/ani14030438

**Published:** 2024-01-29

**Authors:** Maša Vilfan, Urša Lampreht Tratar, Nina Milevoj, Alenka Nemec Svete, Maja Čemažar, Gregor Serša, Nataša Tozon

**Affiliations:** 1 Veterinary Faculty, University of Ljubljana, 1000 Ljubljana, Sloveniaulampreht@onko-i.si (U.L.T.); alenka.nemecsvete@vf.uni-lj.si (A.N.S.); 2 Institute of Oncology Ljubljana, 1000 Ljubljana, Slovenia; mcemazar@onko-i.si (M.Č.); gsersa@onko-i.si (G.S.); 3 Faculty of Health Sciences, University of Primorska, 6310 Izola, Slovenia

**Keywords:** electrochemotherapy, gene electrotransfer, interleukin 12, mast cell tumour, biomarkers, nucleosomes, ferritin, lactate dehydrogenase

## Abstract

**Simple Summary:**

This study focused on identifying and evaluating the use of selected potential biomarkers in predicting the treatment response of mast cell tumours (MCT) in dogs using a combination of electrochemotherapy (ECT) and interleukin 12 gene electrotransfer (IL-12 GET). In the study of 48 patients with 86 MCTs, blood samples were taken before, one month and six months after treatment to identify reliable biomarkers that predict response to treatment. An increased plasma nucleosome concentration and increased serum lactate dehydrogenase (LDH) activity one month after treatment correlated with a more effective local response, such as necrosis and swelling. These biomarkers proved to be potential early indicators of treatment success. However, the serum ferritin concentration did not prove to be meaningful. The results of this study provide crucial insights into the use of the plasma nucleosome concentration and serum LDH activity as potential indicators of treatment efficacy in veterinary oncology. Ultimately, this study emphasises the need to identify and validate predictive biomarkers to improve treatment outcomes in dogs with MCT and other malignancies treated similarly.

**Abstract:**

Electrochemotherapy (ECT) in combination with the gene electrotransfer of interleukin 12 (IL-12 GET) has been successfully used in veterinary medicine for the treatment of mast cell tumours (MCT), but the biomarkers that could predict response to this treatment have not yet been investigated. The aim of this study was to determine the plasma nucleosome and serum ferritin concentrations, as well as the lactate dehydrogenase (LDH) activity, in the serum of treated patients before and one and six months after treatment to evaluate their utility as potential biomarkers that could predict response to the combined treatment. The study was conducted in 48 patients with a total of 86 MCTs that we treated with the combined treatment. The blood samples used for analysing the potential predictive biomarkers were taken before treatment and one and six months after treatment, when the response to treatment was also assessed. The Nu. Q^®^ Vet Cancer Test, the Canine Ferritin ELISA Kit, and the RX Daytona+ automated biochemical analyser were used to analyse the blood samples. The results showed that the plasma nucleosome concentration (before treatment (BT): 32.84 ng/mL (median); one month after treatment (1 M AT): 58.89 ng/mL (median); *p* = 0.010) and serum LDH activity (BT: 59.75 U/L (median); 1 M AT: 107.5 U/L (median); *p* = 0.012) increased significantly one month after treatment and that the increase correlated significantly with the presence of a more pronounced local reaction (necrosis, swelling, etc.) at that time point for both markers (nucleosome: BT (necrosis): 21.61 ng/mL (median); 1 M AT (necrosis): 69.92 ng/mL (median), *p* = 0.030; LDH: BT (necrosis): 54.75 U/L (median); 1 M AT (necrosis): 100.3 U/L (median), *p* = 0.048). Therefore, both the plasma nucleosome concentration and serum LDH activity could serve as early indicators of the effect of the treatment. In this context, the serum ferritin concentration showed no significant predictive potential for treatment response (*p* > 0.999 for all comparisons). In conclusion, this study provides some new and important observations on the use of predictive biomarkers in veterinary oncology. Furthermore, it emphasises the need for the continued identification and validation of potential predictive biomarkers in dogs with MCT and other malignancies undergoing ECT treatment in combination with IL-12 GET to ultimately improve treatment outcomes.

## 1. Introduction

Several studies have been published in which electrochemotherapy (ECT) or a combination of ECT with the gene electrotransfer of interleukin 12 (IL-12 GET) has been successfully used to treat various canine neoplasms such as mast cell tumours (MCT), squamous cell carcinomas, acanthomatous ameloblastomas, perianal tumours and oral malignant melanomas [1,2,3,4,5,6,7]. In human medicine, ECT has proven to be an effective treatment method for various primary malignancies and metastatic diseases. Its application goes beyond the treatment of skin and subcutaneous lesions when combined with imaging and endoscopic procedures, or when used in the treatment of vascular malformations [8,9,10,11].

The basic mechanism of ECT is electroporation, which uses high-voltage pulses to temporarily and reversibly break the cell membrane barrier. This allows larger molecules, such as DNA, to be introduced into the cells. When electroporation is used for the local treatment of tumours by enhancing the toxicity of chemotherapeutic agents, it is called electrochemotherapy. This is an ablative technique that causes the reversible permeability of the cell membrane so that chemotherapeutic agents such as cisplatin and bleomycin can penetrate the tumour cells and cause immunogenic cell death and the activation of the immune cell response [1,6,9,10,12,13]. Recent studies indicate that ECT also has a systemic remote effect on untreated tumours [10,13,14,15,16]. It induces immunogenic cell death, leading to the release of tumour antigens and educating immune cells, resulting in a systemic effect. When ECT is combined with IL-12 GET immunotherapy, which has also shown a systemic remote effect, the overall effect can be enhanced. This enhancement has been demonstrated in preclinical studies in mice, as well as in clinical studies in veterinary and human oncology [1,3,6,15,17,18,19].

Mast cell tumours are the abnormal growth of mast cells. In dogs, MCTs account for 16–21% of all skin neoplasms and can occur in various parts of the body, with cutaneous and subcutaneous sites being the most common [12,20]. Several clinical studies in veterinary oncology have shown that the treatment of various canine cutaneous, subcutaneous and oral tumours, including MCTs, with ECT alone or in combination with IL-12 GET results in a high percentage of long-lasting objective responders (i.e., animals that had a complete or partial response to treatment, according to RECIST criteria [21]) [1,3,6,22]. Currently, decision making regarding treatment in ECT practice is guided by clinical factors such as the tumour histiotype, tumour size and previous treatments, while there are no known biomarkers that could predict the response to treatment [23].

Biomarkers are defined as indicators of normal biological processes, pathogenic processes or responses to an exposure or intervention, including therapeutic interventions. Based on their clinical use, biomarkers can be categorised as susceptibility or risk, diagnostic, monitoring, prognostic, predictive, pharmacodynamic or response and safety biomarkers [24]. Currently, the most commonly used method for the identification and analysis of biomarkers in oncology is tumour biopsy [23]. As biopsies can be invasive and expensive, and can increase the risk of adverse events, the search for biomarkers in peripheral blood for clinical utility is the next logical step [25]. Biomarkers that could predict the response to treatment of different tumours with ECT or the combination of ECT with IL-12 GET have not yet been investigated in veterinary oncology. Nucleosome and ferritin concentrations and the LDH activity in blood have been shown to be valuable diagnostic biomarkers for various malignancies in animals [26,27,28,29,30,31,32,33,34,35].

Nucleosomes are important components of chromosomes and consist of DNA wrapped around nuclear histones. They perform several functions, including condensing chromatin, protecting DNA, and regulating genes by restricting the binding of transcription factors. While nucleosomes are present in all mammalian cells, they can also be found in the bloodstream and are normally released by activated or dying white blood cells [36]. Severe inflammation or trauma results in the significant release of nucleosomes [37,38,39]. Elevated nucleosome concentrations have been observed in humans and dogs in different malignancies, suggesting that nucleosomes may serve as biomarkers for the early detection of the disease [35,36,40,41,42]. In addition, nucleosomes, their core components histones, and other proteins are the integral constituents of liquid biopsies, a new approach to cancer detection and monitoring [43,44].

Ferritin, a protein involved in intracellular iron storage and cell protection, is found in high concentrations in the liver, spleen, and bone marrow of mammals [45,46,47]. High serum ferritin levels are influenced by conditions such as iron overload, inflammatory diseases, liver damage and malignant tumours [48]. Malignant tumours have been shown to produce and release ferritin, leading to an increase in serum ferritin levels [26,49]. Elevated serum ferritin levels have also been observed in clinical studies in tumour patients and have been attributed to reduced iron utilisation during tumour development [29,50]. In veterinary medicine, elevated serum ferritin concentrations are observed in dogs with lymphomas, histiocytic sarcomas, mammary tumours, immune-mediated haemolytic anaemia (IMHA) and liver disease [26,27,28,29].

Lactate dehydrogenase (LDH), present in five isoenzymes, is a ubiquitous glycolytic enzyme involved in the metabolism of glucose and the production of lactate [51]. The production of lactate plays a significant role in the malignant progression of cancer by facilitating tumour cell migration and invasion, promoting angiogenesis, and causing the suppression of immunity [52]. Elevated serum LDH activity is thought to reflect high metabolic activity in the hypoxic and highly active cellular environment of malignant tumours [30,31]. Studies in human and veterinary medicine have shown a significant increase in serum LDH activity in patients with lymphomas, leukaemias, mammary gland tumours and oral cavity tumours, especially oral malignant melanoma [30,31,32,33,34].

As far as the authors are aware, the relationship between the effectiveness of treatment with ECT and IL-12 GET and the levels of nucleosomes, ferritin or LDH has not yet been studied in veterinary medicine. The aim of this study was therefore to investigate the possible changes in the plasma nucleosome and serum ferritin concentrations, as well as in the serum LDH activity, before and after the combined treatment of MCT with ECT and IL-12 GET in dogs in relation to the clinical response.

## 2. Materials and Methods

This study included 48 dogs (Table 1) that presented to the Small Animal Clinic of the Veterinary Faculty in Ljubljana with 86 MCTs and were enrolled in a clinical study entitled “Monitoring of clinical and immune response to improve the outcome of combined ECT and IL-12 GET in dogs with spontaneous peripheral tumours” between April 2021 and July 2023. The clinical trial was approved by the Ethics Committee of the Ministry of Agriculture, Forestry, and Food of the Republic of Slovenia (approval no. 254-20/2015/7, and permit for the deliberate release of genetically modified organisms 35419/2021-2550-8). All dogs included in this study met the inclusion criteria, i.e., they had to have at least one cytologically or histopathologically diagnosed peripheral (cutaneous, subcutaneous or mucosal) MCT and an anticipated life expectancy of more than three months. Patients who had received systemic chemotherapy or tyrosine kinase inhibitors (masitinib or toceranib) in the previous 14 days and patients with evidence of other systemic disease (renal insufficiency, cardiovascular disease) at the time of inclusion were excluded. Pregnancy or lactation were also excluding criteria. The determination of whether other systemic diseases were present was based on a comprehensive assessment that included the patients’ medical history, clinical examination findings, haematological and biochemical analysis results and, if indicated, diagnostic imaging. Written informed consent was obtained from the owner of each animal prior to participation in the clinical trial.

Disease staging was performed at inclusion and consisted of a clinical examination of the patient, a basic haematological and biochemical examination, and an assessment of the size and growth pattern of each MCT and the number of MCTs present. In addition, the regional lymph nodes were palpated, and a fine needle biopsy and cytology were performed on the liver and spleen and on suspicious lymph nodes if clinically indicated. The samples for cytological and pathohistological analysis were forwarded to the Laboratory for Pathoanatomical Diagnostics and Pathohistology of the Veterinary faculty in Ljubljana. The samples were analysed by one of the non-board-certified experienced pathologists on duty.

Based on this information, animals were categorised into five stages, in accordance with the proposed amendment to the WHO clinical staging for canine MCT [53]. In patients where tumour biopsy and histopathology were performed, MCT was graded according to both the Kiupel and Patnaik schemes [54,55]. The sample used for pathohistological grading was a four-millimetre punch biopsy (MedGyn Disposable Punch 4 mm. MedGyn, Addison, IL, USA) that was performed prior to treatment. In cases where an animal had several tumours, only one tumour was preferably sampled, with the largest tumour usually selected for sampling and grading. Furthermore, blood samples were taken from the included dogs at various time points to determine the plasma nucleosome and serum ferritin concentrations, as well as the serum LDH activity.

**Table 1:** Demographic data for included patients.

Patient Number	Breed	Sex	Age [Years]	Site of the Tumours	Histopathological Grade *	Clinical Stage **	Longest Diameter of the Tumour [cm] (Tumor Volume)
1	Boxer	F	6.3	Left hind leg	ND	I	2.2 (3.63 cm^3^)
2	Miniature Schnauzer	F	11.3	Left front leg	ND	I	2.1 (2.78 cm^3^)
3	Crossbreed	F	7.7	Left front leg	ND	I	2.3 (4.12 cm^3^)
4	Boxer	F	9.8	Right inguinal region	II (low)	I	4.0 (17.42 cm^3^)
5	Boston Terrier	M	10.4	Right hind leg	ND	II	1.5 (0.78 cm^3^)
				Right scapula			0.7 (0.11 cm^3^)
				Left hind leg			1.9 (3.40 cm^3^)
				Left inguinal region			0.5 (0.07 cm^3^)
6	French Bulldog	F	10.0	Right hind leg	II (low)	I	1.6 (1.09 cm^3^)
7	Golden Retriever	F	3.4	Left scapula	ND	I	1.2 (0.76 cm^3^)
8	Crossbreed	F	11.1	Right dorsum	ND	I	1.7 (1.49 cm^3^)
9	Crossbreed	F	9.9	Left hind leg	ND	I	2.0 (2.93 cm^3^)
10	Dogo Argentino	F	6.8	Right front leg	ND	I	3.8 (13.72 cm^3^)
11	Dachshund	M	11.0	Left ear	ND	II	0.5 (0.07 cm^3^)
				Dorsal part of the neck			0.5 (0.07 cm^3^)
				Right abdominal region			0.5 (0.07 cm^3^)
				Perianal region			0.8 (0.17 cm^3^)
12	Crossbreed	M	10.2	Prepuce	I (low)	I	0.5 (0.07 cm^3^)
13	Jack Russell Terrier	M	7.8	Left front leg	II (low)	I	5.0 (0.05 cm^3^)
				Right lip commissure			0.8 (0.27 cm^3^)
14	Crossbreed	F	6.6	Dorsum	ND	II	0.4 (0.33 cm^3^)
				Left abdominal region			0.4 (0.33 cm^3^)
				Left hind leg			0.5 (0.07 cm^3^)
				Left hind leg			0.5 (0.07 cm^3^)
				Tail base			1.1 (0.70 cm^3^)
				Vulva			0.4 (0.33 cm^3^)
15	Boston Terrier	F	7.2	Left elbow	ND	I	1.2 (0.53 cm^3^)
16	Manchester Terrier	F	8.2	Right inguinal region	ND	I	2.8 (3.62 cm^3^)
17	Golden Retriever	F	7.7	Chin	II (low)	II	1.5 (0.78 cm^3^)
				Right thoracic region			1.0 (0.52 cm^3^)
				Right hind leg			0.5 (0.04 cm^3^)
				Right hind leg			0.6 (0.09 cm^3^)
18	English Greyhound	M	4.7	Perianal region	II (low)	I	1.4 (0.88 cm^3^)
19	Greater Swiss Mountain Dog	F	3.3	Left hind leg	II (low)	I	2.3 (3.71 cm^3^)
20	Boston Terrier	M	8.9	Left abdominal region	ND	II	2.4 (1.32 cm^3^)
				Left front leg			0.5 (0.07 cm^3^)
				Right hind leg			2.2 (0.69 cm^3^)
21	Shih Tzu	M	6.5	Right hind leg	II (low)	I	2.0 (1.63 cm^3^)
22	French Bulldog	M	6.0	Right orbital region	II (low)	II	0.8 (0.20 cm^3^)
				Perianal region			0.7 (0.07 cm^3^)
				Perianal region			1.3 (0.12 cm^3^)
				Right hind leg			0.3 (0.01 cm^3^)
23	Boston Terrier	M	6.1	Right front leg	ND	II	1.0 (0.42 cm^3^)
				Left front leg			0.5 (0.07 cm^3^)
				Sternal region			0.5 (0.07 cm^3^)
				Right orbital region			0.5 (0.07 cm^3^)
24	Newfoundland	M	8.6	Right hind leg	ND	I	1.3 (0.60 cm^3^)
25	Boston Terrier	M	8.4	Prepuce	II (low)	II	1.1 (0.06 cm^3^)
				Right hind leg			1.1 (0.02 cm^3^)
				Perianal region			1.2 (0.28 cm^3^)
				Right abdominal region			1.1 (0.23 cm^3^)
26	French Bulldog	M	6.2	Prepuce	II (low)	I	1.5 (1.12 cm^3^)
27	Hungarian Vizsla	F	9.9	Right ear	II (low)	I	1.2 (0.28 cm^3^)
28	Boxer	M	5.8	Left ear	II (low)	I	1.1 (0.29 cm^3^)
29	Pit bull	M	8.3	Left abdominal region	II (low)	I	1.5 (0.55 cm^3^)
30	Crossbreed	F	12.2	Left thoracic region	ND	II	0.5 (0.07 cm^3^)
				Left thoracic region			0.5 (0.07 cm^3^)
				Left hind leg			0.5 (0.07 cm^3^)
				Right ear			0.2 (0.004 cm^3^)
				Right thoracic region			0.5 (0.07 cm^3^)
				Left inguinal region			0.5 (0.07 cm^3^)
31	Havanese	F	9.3	Left hind leg	II (low)	I	1.0 (0.25 cm^3^)
32	Maltese	F	10.8	Tail	ND	I	0.9 (0.20 cm^3^)
33	Boston Terrier	F	8.4	Right front leg	ND	I	1.8 (1.60 cm^3^)
34	French Bulldog	M	8.7	Left hind leg	II (low)	II	1.7 (1.99 cm^3^)
				Left hind leg			0.7 (0.18 cm^3^)
				Scrotum			0.9 (0.38 cm^3^)
				Prepuce			0.9 (0.38 cm^3^)
				Left front leg			0.7 (0.18 cm^3^)
35	French Bulldog	M	5.5	Prepuce	II (low)	I	1.0 (0.37 cm^3^)
				Right hind leg			0.8 (0.27 cm^3^)
36	Boxer	M	8.3	Right front leg	II (low)	I	4.0 (8.29 cm^3^)
37	Boxer	M	7.6	Right abdominal region	II (low)	I	1.5 (1.53 cm^3^)
				Perianal region			2.0 (3.77 cm^3^)
38	Boxer	F	8.8	Right lip corner	Subcutaneous	I	1.5 (1.12 cm^3^)
39	Yorkshire Terrier	F	13.6	Left front leg	ND	I	1.1 (0.06 cm^3^)
				Left front leg			1.1 (0.02 cm^3^)
40	Crossbreed	F	10.7	Frontal region	ND	III	2.5 (3.14 cm^3^)
41	Boxer	M	5.2	Left ear	II (low)	I	1.0 (0.33 cm^3^)
42	Boxer	F	6.4	Left hind leg	Subcutaneous	I	2.2 (3.29 cm^3^)
43	French Bulldog	F	2.5	Perianal region	II (low)	I	1.0 (0.42 cm^3^)
44	Boxer	M	4.1	Right hind leg	II (low)	I	1.0 (0.42 cm^3^)
45	Crossbreed	F	11.5	Left axillary region	II (high)	III	3.7 (14.33 cm^3^)
46	Crossbreed	F	7.0	Right hind leg	Subcutaneous	III	2.0 (2.35 cm^3^)
47	Maltese	F	7.4	Right lip commissure	MCT mucosae labie	I	1.5 (0.47 cm^3^)
48	Boston Terrier	F	7.4	Right hind leg	II (high)	I	1.8 (1.27 cm^3^)

M, male; F, female; ND, not determined; * grade according to the Patnaik (Kiupel) system [54,55]; ** clinical stage according to the proposed amendment to the WHO staging system for MCTs [53].

### 2.1. Blood Sampling and Processing

The blood samples to be used for serum and plasma preparation were collected from the included patients before the start of therapy and four weeks after therapy. The collection of samples at six months post treatment depended on the treatment response and possible re-treatment, owner compliance and the time of inclusion in this study. At each sampling, we collected five millilitres of venous blood into two micro sample tubes containing the anticoagulant sodium citrate (Sarstedt AG & Co. KG, Nümbrecht, Germany) and one serum separation tube (Jørgen Kruuse A/S, Langeskov, Denmark). After centrifugation, the serum and plasma were harvested and aliquoted. The serum samples were stored at −20 °C and the plasma samples at −80 °C until later batch analyses.

### 2.2. Treatment Protocol

All dogs were treated with a combination of ECT and IL-12 GET using the previously described plasmid for canine IL-12 without the antibiotic resistance gene (pORFcaIL-12-ORT, COBIK, Ajdovscina, Slovenia) [56]. On the day of treatment, they were examined by an anaesthesiologist and placed under general anaesthesia as previously described [3]. An abdominal ultrasound with an ultrasound-guided fine needle biopsy of the spleen and liver was performed as part of the staging of the disease. The cytostatic drug used in the treatment protocol was either cisplatin (Cisplatin Accord, Accord Health Care, London, UK) or bleomycin (Bleomycin Medac, Medac GmbH, Hamburg, Germany). Cisplatin was the first-line cytostatic drug, but replaced by bleomycin if multiple tumours were present, if the tumour was ulcerated or bleeding, or if the animal had been previously treated with cisplatin and did not respond adequately [6]. The treated tumours were measured in three dimensions using a calliper and the tumour volume was calculated using the following formula: V = a × b × c × π/6 [3]. When cisplatin (1 mg/mL) was used, the drug was administered intratumorally (i.t.) one minute before electroporation at a dose of 1 mg/cm^3^ for tumours smaller than 1 cm^3^ and 0.5 mg/cm^3^ for tumours larger than 1 cm^3^. When bleomycin was used, the drug was dissolved at a concentration of 3 mg/mL and administered intravenously at a dose of 0.3 mg/kg eight minutes before electroporation [6]. Plasmid DNA with IL-12 was administered intratumorally five minutes before electroporation, regardless of the cytostatic drug chosen. An electroporation protocol with eight electrical pulses, a 100 µs duration, a 1300 V/cm ratio and a 5 kHz frequency, was performed using the Cliniporator™ pulse generator (IGEA s.r.l., Carpi, Italy). Depending on the tumour size, stainless steel plate electrodes (6 mm spacing), needle electrodes (4 mm spacing) or hexagonal electrodes were used. The delivery of a sequential pulse from the tumour edge towards the centre reduced the blood flow, resulting in vascular lock and the better retention of the chemotherapeutic agent in the tumour nodule [57].

### 2.3. Evaluation of Treatment Outcome

**Figure 1:**
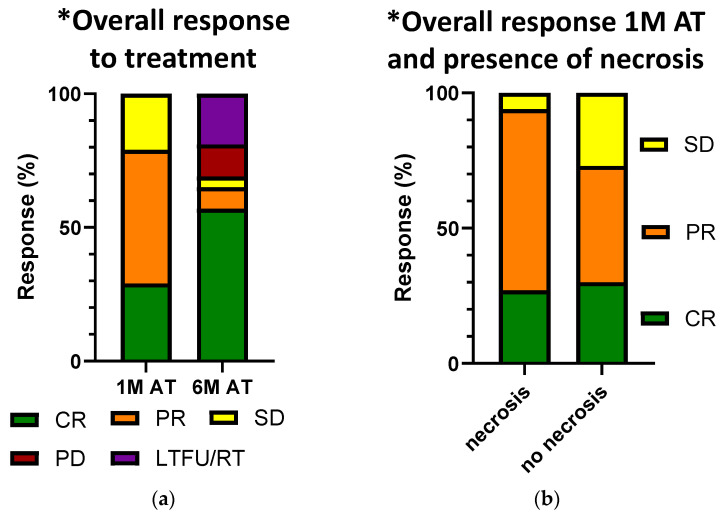
(**a**) **Comparison of overall responses to treatment one month post treatment and six months post treatment**. Responses were evaluated according to the RECIST criteria [21]. The graph shows a high overall response rate (CR and PR combined) one month after treatment. At six months after treatment, this rate further increases, as well as the percentage of CR, when excluding the LTFU/RT patients. CR, complete response; PR, partial response; SD, stable disease; PD, progressive disease; LTFU/RT, lost to follow-up/re-treatment; 1 M AT, one month after treatment; 6 M AT, six months after treatment. * In patients with more than one MCT, we only recorded the least favourable response to treatment; (**b**) **correlation of overall response to treatment and presence of necrosis one month post treatment.** The graph shows a higher percentage for the overall response rate (CR and PR combined) in patients where necrosis was seen at one month post treatment. CR, complete response; PR, partial response; SD, stable disease; PD, progressive disease; LTFU/RT, lost to follow-up/re-treatment; 1 M AT, one month after treatment. * In patients with more than one MCT, we only recorded the least favourable response to treatment.

After the treatment, the follow-up visits were scheduled at one week, four weeks, two months, three months, and six months after the treatment, based on previously described operating procedures [57]. At each visit, the dogs were clinically examined, the treatment site was thoroughly inspected, any residual tumours were measured with a calliper and the treatment site was photographed. The owners were interviewed about the dog’s general well-being and response to therapy. In line with the protocol described above, blood was drawn again four weeks after therapy, in some patients also six months after therapy (Table 2), and stored accordingly. However, patients who did not respond satisfactorily to their initial treatment and required re-treatment during the observation period were excluded from sampling at the six-month follow up. In some cases, owners declined the six-month follow-up due to a positive response to treatment and considered the appointment unnecessary. In addition, patients enrolled in the study after February 2023 were not sampled at the six-month follow-up because the batch and statistical analyses were completed in July 2023, which was prior to their scheduled six-month follow-up. The patients’ response to therapy was determined according to the RECIST criteria (Table 3): complete response (CR), i.e., disappearance of the tumour; partial response (PR), i.e., reduction in the tumour by at least 30%; stable disease (SD), i.e., reduction in the tumour by less than 30% or enlargement by less than 20%; and progressive disease (PD), i.e., enlargement of the tumour by at least 20% [21]. Some of the patients (15/48) reacted more conspicuously to the treatment than others, with the occurrence of tissue swelling, redness, an open wound or enhanced tumour necrosis in the treated area, which may have been due to the inflammatory process triggered by the combined treatment. This response was observed and visually assessed by one of three veterinarians that were conducting the clinical examination of the treated animals in this study. When assessing the response to treatment in patients with multiple treated tumours, the response was recorded for each individual tumour site. The least favourable response was considered to determine the overall response for the assessment of potential biomarkers that may correlate with the presence of disease (Figure 1a). This approach is based on the consideration that if one of the treated sites still shows signs of the disease, this could have a systemic impact on the potential biomarkers tested in the blood.

### 2.4. Sample Analysis

#### 2.4.1. Nucleosomes and Ferritin

The concentrations of nucleosomes and ferritin were determined from the plasma (nucleosomes) and serum (ferritin) samples using the Nu. Q^®^ Vet Cancer Test (Belgian Volition SRL, Isnes, Belgium), a sandwich format assay for the determination of nucleosomes, and the Canine Ferritin ELISA Kit (MyBioSource, Inc., San Diego, CA, USA) for ferritin determination, according to the manufacturer’s instructions. In brief, samples were placed in wells coated with anti-H3.1 nucleosome monoclonal antibody or anti-Canine FE monoclonal antibody. The horseradish peroxidase (HRP) system was used for detection. Absorbance measurements were used to determine the concentration of H3.1 nucleosomes and ferritin in the samples.

#### 2.4.2. Lactate Dehydrogenase

The lactate dehydrogenase activity was measured spectrophotometrically using an RX Daytona+ automated biochemical analyser (Randox, Crumlin, UK) and the Randox lactate dehydrogenase L-lactate–Pyruvate reagent kit (Randox). A brief description of the assay principle is as follows: Nicotinamide adenine dinucleotide (NAD+) and lactate are converted to pyruvate and NADH (reduced form of NAD+) at the same rate in equimolar amounts. The rate at which NADH is formed is determined by an increase in the absorbance and is directly proportional to the LDH activity. The normal range for LDH at the laboratory in which analysis was preformed is 21–217 U/L.

### 2.5. Statistical Analysis

Statistical analysis was performed using GraphPad Prism 10 (Systat software, London, UK). A Shapiro–Wilk test was performed to determine the distribution of the data. The data were not normally distributed; therefore, significance was determined using a Kruskal–Wallis or Wilcoxon test; *p* < 0.05 values were considered statistically significant. The values were expressed as medians with quartile 1 and quartile 3. The groups that were compared in the statistical analysis for ferritin, nucleosomes and LDH were marked as before treatment (BT), one month after treatment (1 M AT) and six months after treatment (6 M AT).

## 3. Results

In this study, we included 48 dogs with 86 MCTs treated with a combination of ECT and GET with pORFcaIL-12-ORT. We followed the patients for six months after treatment and analysed the levels of plasma and serum biomarkers.

### 3.1. The Percentage of Complete Responses (CR) after the Combination of ECT and IL-12 GET Increased after 6 Months

The antitumour effectiveness was evaluated one and six months after treatment (Figure 1a). The results showed that one month after treatment, the overall response rate (ORR) was 83.7%, of which 43.0% was CR and 40.7% was PR. Six months after treatment, the percentage of CR increased to 76.8% and PR changed to 11.6%, with an ORR of 88.4%, similar to observations in previously published results [6,58,59]. Seventeen tumours in eight patients could not be evaluated at six months after treatment because they had undergone repeated treatment or another treatment or were lost to follow-up (Table 3). Therefore, they were excluded from the six-month statistics. No PD was observed one month after treatment, but six months after treatment, PD was seen in five tumours (7.2%).

### 3.2. The Nucleosome Concentration Significantly Increased One Month after Treatment. Enhanced Tumour Necrosis Was Positively Associated with Elevations in Nucleosome Concentration One Month after Treatment

Plasma for the measurement of the nucleosome concentration was collected from 43 dogs in this study. The Nu. Q^®^ Vet Cancer Test has a standardised reference range for plasma H3.1 nucleosome concentrations. It categorises the concentrations as high range (>67.4 ng/mL), grey zone (>30.4 and ≤67.4 ng/mL) or low range (≤30.4 ng/mL). In our study, we observed that before treatment, 23.3% of the included patients (10/43) had high concentrations of nucleosomes, 27.9% (12/43) were in the grey zone and 48.8% (21/43) were in the low range.

**Figure 2:**
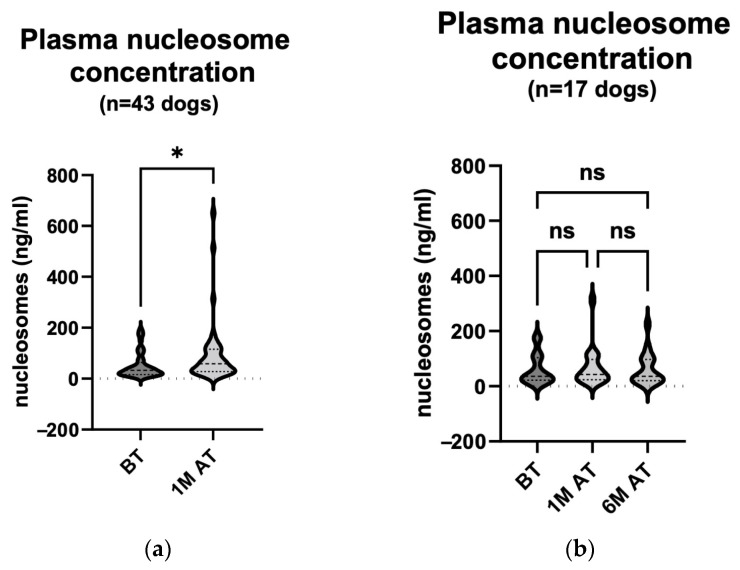
(**a**) **Plasma nucleosome concentration comparison: pre**-**treatment and one**-**month post-treatment in 43 dogs**. The concentration was measured using the Nu. Q^®^ Vet Cancer Test on the day of the treatment and four weeks later. The analysis showed a statistically significant rise in the plasma nucleosome concentration one month following the treatment (*p* = 0.010). BT, before treatment; 1 M AT, one month after treatment * *p* < 0.05. (**b**) **Plasma nucleosome concentration comparison: pre**-**treatment, one month post treatment and six months post treatment in 17 dogs**. The concentration was measured using the Nu. Q^®^ Vet Cancer Test on the day of the treatment, four weeks later and at the end of the observation period. The analysis showed no statistically notable changes in the plasma nucleosome concentration. BT, before treatment; 1 M AT, one month after treatment; 6 M AT, six months after treatment; ns, not significant (*p* > 0.999 for all three comparisons).

We then compared the results of the samples collected before and one month after treatment (Figure 2a). The results showed a statistically significant increase (*p* = 0.010) in the plasma nucleosome concentration one month after the treatment (median value 32.84 ng/mL (15.87–64.07 ng/mL) before treatment and 58.89 ng/mL (28.24–115.2 ng/mL) one month after treatment) of the tumours with a combination of ECT and IL-12 GET. Six months after treatment, only 17 patients were re-sampled for analysis (Table 2). When we compared the results before treatment (median 36.64 ng/mL (21.89–103.6 ng/mL)), one month after treatment (median 42.54 ng/mL (23.97–97.39 ng/mL)) and six months after treatment (median 36.07 ng/mL (20.00–96.90 ng/mL)), there were no statistically significant changes (*p* > 0.999) in the relevant comparisons (Figure 2b). When comparing only the results of the 17 dogs that were also sampled six months after treatment, the statistically significant increase in the plasma nucleosome concentration previously observed was no longer significant in a smaller number of patients.

**Figure 3:**
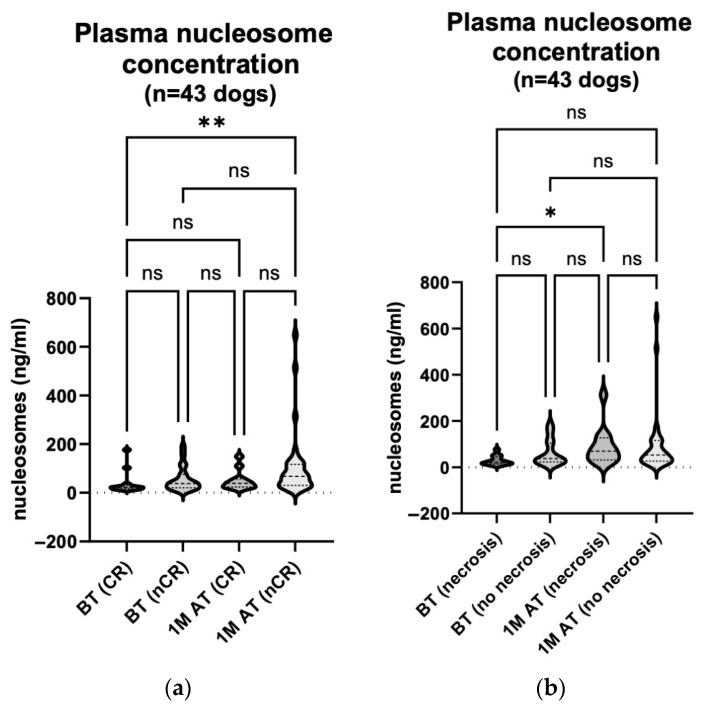
(**a**) **Plasma nucleosome concentration comparison: pre**-**treatment and one month post treatment in correlation with response to treatment in 43 dogs.** The concentration was measured using the Nu. Q^®^ Vet Cancer Test on the day of treatment and four weeks later. The analysis showed no statistically remarkable changes in the plasma nucleosome concentration (*p*: BT (CR) vs. BT (nCR) = 0.624; BT (CR) vs. 1 M AT (CR) = 0.965; BT (CR) vs. 1 M AT (nCR) = 0.008; BT (nCR) vs. 1 M AT (CR) > 0.999; BT (nCR) vs. 1 M AT (nCR) = 0.195; 1 M AT (CR) vs. 1 M AT (nCR) = 0.741). BT, before treatment; 1 M AT, one month after treatment; CR, complete response; nCR, non-complete response (comprising of PR, SD, and PD); ns, not significant ** *p* < 0.01. (**b**) **Plasma nucleosome concentration comparison: pre**-**treatment and one month post treatment in correlation with treatment**-**related enhanced tumour necrosis in 43 dogs.** The concentration was measured using the Nu. Q^®^ Vet Cancer Test on the day of treatment and four weeks later. The analysis showed a statistically remarkable rise in the plasma nucleosome concentration one month after treatment in patients where tumour necrosis was seen. BT, before treatment; 1 M AT, one month after treatment; ns, not significant (*p*: BT (necrosis) vs. BT (no necrosis) = 0.275; BT (necrosis) vs. 1 M AT (necrosis) = 0.030; BT (necrosis) vs. 1 M AT (no necrosis) = 0.055; BT (no necrosis) vs. 1 M AT (necrosis) > 0.999; BT (no necrosis) vs. 1 M AT (no necrosis) > 0.999; 1 M AT (necrosis) vs. 1 M AT (no necrosis) > 0.999); * *p* < 0.05.

One month after the treatment of the MCTs, 12 of the 43 dogs in which the plasma nucleosome level was determined had a CR (median 21.89 ng/mL (15.00–33.17 ng/mL) before treatment and median 38.34 ng/mL (24.13–57.00 ng/mL) one month after treatment). In the remaining 31 animals, the response was either PR, SD, or PD (median 38.23 ng/mL (20.14–76.16 ng/mL) before treatment and median 67.83 ng/mL (30.00–117.2 ng/mL) one month after treatment). The latter three responses were grouped as a non-complete response (nCR) for comparison with the CR group. We found no statistically significant changes between the respective groups (Figure 3a; *p*: BT (CR) vs. BT (nCR) = 0.624; BT (CR) vs. 1 M AT (CR) = 0.965; BT (CR) vs. 1 M AT (nCR) = 0.008; BT (nCR) vs. 1 M AT (CR) > 0.999; BT (nCR) vs. 1 M AT (nCR) = 0.195; 1 M AT (CR) vs. 1 M AT (nCR) = 0.741).

Similarly, we compared the plasma nucleosome levels before and one month after treatment in the 12 dogs that responded with the enhanced necrosis of tumour tissue (median 21.61 ng/mL (15.12–45.86 ng/mL) before treatment and 69.92 ng/mL (31.94–127.2 ng/mL) one month after treatment) and in the 31 that showed no signs of necrosis (median 38.23 ng/mL (23.82–104.2 ng/mL) before treatment and 52.64 ng/mL (27.44–115.2 ng/mL) one month after treatment). In the first group, the nucleosome levels were statistically significantly elevated one month after treatment (Figure 3b; *p*: BT (necrosis) vs. BT (no necrosis) = 0.275; BT (necrosis) vs. 1 M AT (necrosis) = 0.030; BT (necrosis) vs. 1 M AT (no necrosis) = 0.055; BT (no necrosis) vs. 1 M AT (necrosis) > 0.999; BT (no necrosis) vs. 1 M AT (no necrosis) > 0.999; 1 M AT (necrosis) vs. 1 M AT (no necrosis) > 0.999).

### 3.3. Ferritin Concentrations Did Not Correlate with Time before or after Treatment, nor with Treatment Efficacy

**Figure 4:**
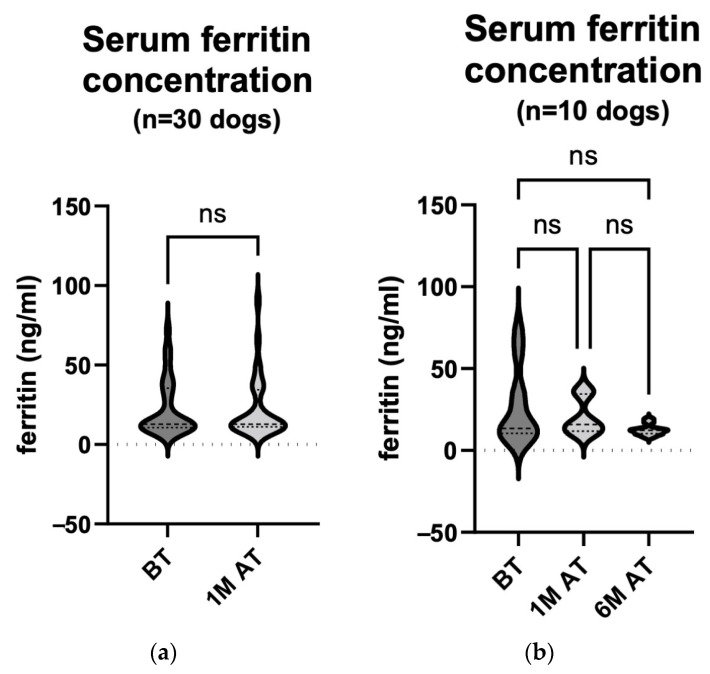
(**a**) **Serum ferritin concentration comparison: pre**-**treatment and one month post treatment in 30 dogs.** The concentration was measured using the Canine Ferritin ELISA Kit on the day of the treatment and four weeks later. The analysis showed no statistically significant change in the serum ferritin concentration one month following the treatment (*p* = 0.667). BT, before treatment; 1 M AT, one month after treatment; ns, not significant. (**b**) **Serum ferritin concentration comparison: pre**-**treatment, one month post treatment and six months post treatment in 10 dogs.** The concentration was measured using the Canine Ferritin ELISA Kit on the day of the treatment, four weeks later and at the end of the observation period. The analyses showed no statistically significant changes in the serum ferritin concentrations (*p*: BT vs. 1 M AT > 0.999; BT vs. 6 M AT = 0.221; 1 M AT vs. 6 M AT = 0.076). BT, before treatment; 1 M AT, one month after treatment; 6 M AT, six months after treatment; ns, not significant.

The serum ferritin concentration was measured in 30 dogs before and one month after treatment. Samples were also taken from 10 of the 30 dogs six months after treatment (Table 2). We compared the serum ferritin concentration before treatment (median 12.75 ng/mL (10.59–35.46 ng/mL)) and one month after treatment (median 12.70 ng/mL (11.13–34.39 ng/mL)) in all 30 dogs, regardless of the treatment response (Figure 4a; *p* = 0.667). We also compared 10 of the 30 animals before treatment (median 13.37 ng/mL (10.43–41.33 ng/mL)), one month (median 15.78 ng/mL (11.74–34.39 ng/mL)) and six months after treatment (median 12.24 ng/mL (10.26–13.95 ng/mL)). Neither comparison showed statistically significant changes in the serum ferritin concentrations (Figure 4b; *p*: BT vs. 1 M AT > 0.999; BT vs. 6 M AT = 0.221; 1 M AT vs. 6 M AT = 0.076).

**Figure 5:**
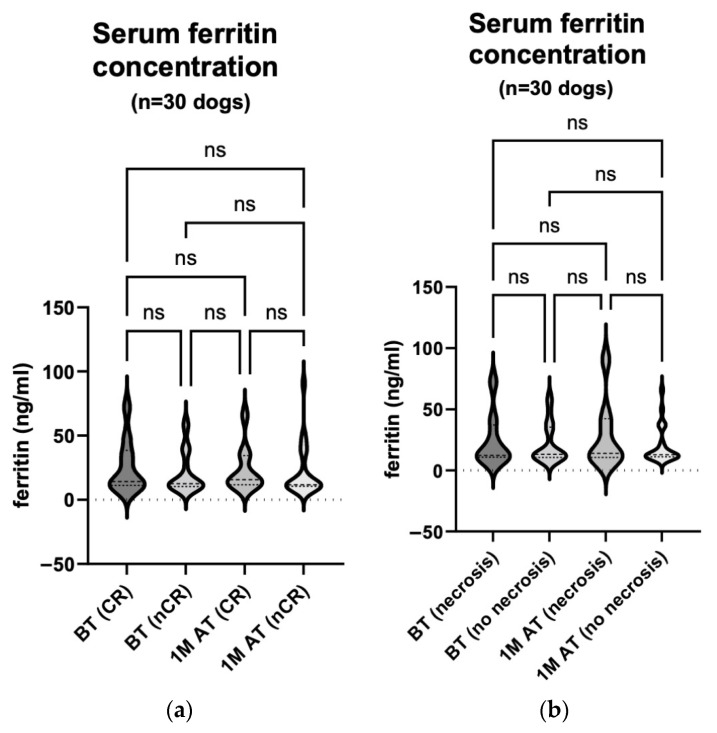
(**a**) **Serum ferritin concentration comparison: pre**-**treatment and one month post treatment in correlation with response to treatment in 30 dogs.** The concentration was measured using the Canine Ferritin ELISA Kit on the day of treatment and four weeks later. The analyses showed no statistically remarkable changes in the serum ferritin concentrations (*p* > 0.999 for all comparisons). BT, before treatment; 1 M AT, one month after treatment; CR, complete response; nCR, non-complete response (comprising of PR, SD, and PD); ns, not significant. (**b**) **Serum ferritin concentration comparison: pre**-**treatment and one month post treatment in correlation with treatment- related enhanced tumour necrosis in 30 dogs.** The concentration was measured using the Canine Ferritin ELISA Kit on the day of treatment and four weeks later. The analyses showed no statistically remarkable changes in the serum ferritin concentrations (*p* > 0.999 for all comparisons). BT, before treatment; 1 M AT, one month after treatment; ns, not significant.

In addition, we compared the serum ferritin concentrations measured before treatment and one month after treatment between the animals with CR (median 14.33 ng/mL (11.19–38.44 ng/mL) before treatment and median 15.78 ng/mL (11.68–34.39 ng/mL) one month after) and the animals whose response to treatment was classified as PR, SD or PD (median 12.52 ng/mL (10.46–33.55 ng/mL) before treatment and median 11.71 ng/mL (10.65–33.40 ng/mL) one month after treatment). The latter three responses were grouped as a non-complete response (nCR) for comparison with the CR group. These comparisons did not yield statistically significant results (Figure 5a; *p* > 0.999 for all comparisons).

The enhanced necrosis of tumour tissue at the treatment site was noted in eight of the 30 dogs. We compared the serum ferritin concentrations of these 8 dogs before treatment (median 12.32 ng/mL (10.70–37.22 ng/mL)) and one month after treatment (median 13.94 ng/mL (10.56–42.34 ng/mL)) with those of the remaining 22 dogs before treatment (median 13.13 ng/mL (10.59–35.46 ng/mL)) and one month after treatment (median 12.70 ng/mL (11.13–25.85 ng/mL)); this was in order to determine whether enhanced necrosis affected the release of serum ferritin. These comparisons also revealed no statistically significant changes (Figure 5b; *p* > 0.999 for all comparisons).

### 3.4. LDH Activity Increased Significantly Four Weeks after Treatment in All Patients and in Those with Treatment-Related Enhanced Tumour Necrosis

**Figure 6:**
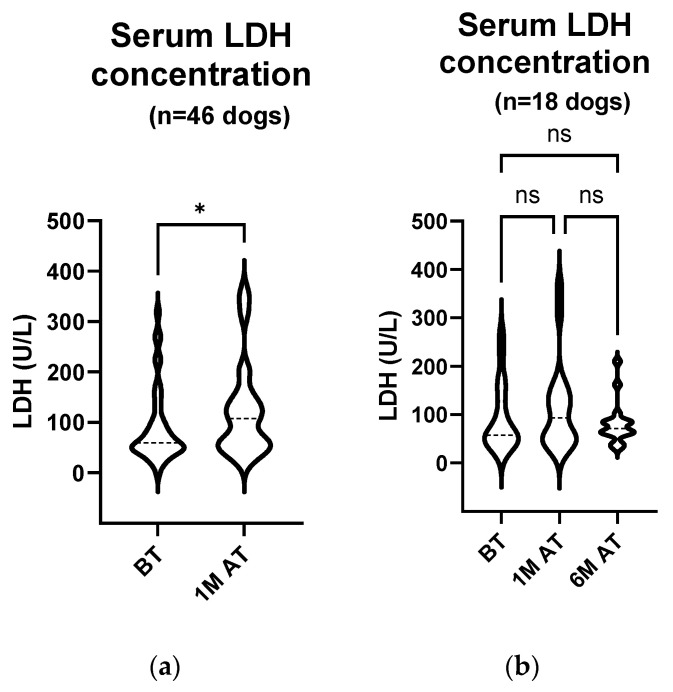
(**a**) **Serum LDH activity comparison: pre**-**treatment and one month post treatment in 46 dogs.** The enzyme activity was measured with an automated biochemical analyser on the day of treatment and four weeks later. The analyses showed a statistically significant increase in the serum LDH activity one month after treatment (*p* = 0.012). BT, before treatment; 1 M AT, one month after treatment; * *p* < 0.05. (**b**) **Serum LDH activity comparison: pre**-**treatment, one month post treatment and six months post treatment in 18 dogs.** The enzyme activity was measured on the day of treatment, four weeks later and at the end of the observation period using an automated biochemical analyser. The analyses showed no statistically remarkable changes in the serum LDH activity (*p* > 0.999 for all comparisons). BT, before treatment; 1 M AT, one month after treatment; 6 M AT, six months after treatment; ns, not significant.

Serum from 46 dogs that participated in the study was collected before and one month after treatment to measure the LDH activity (Figure 6a). In total, 5 out of 46 dogs or 10.8% had elevated serum LDH activity before treatment. A comparison of the two groups showed a statistically significant increase in the serum LDH activity one month after treatment (median 59.75 U/L (45.75–105.0 U/L) before treatment and median 107.5 U/L (54.38–145.9 U/L) one month after treatment; *p* = 0.012). Samples were taken again from 18 of the 46 dogs six months after treatment (Table 2). When comparing the LDH concentrations of only these 18 animals before treatment (median 57.75 U/L (47.25–143.0 U/L)), one month (median 93.25 U/L (48.38–145.9 U/L)) and six months after treatment (median 71.00 U/L (61.63–86.63 U/L)), no statistically significant change was observed (Figure 6b; *p* > 0.999 for all comparisons).

**Figure 7:**
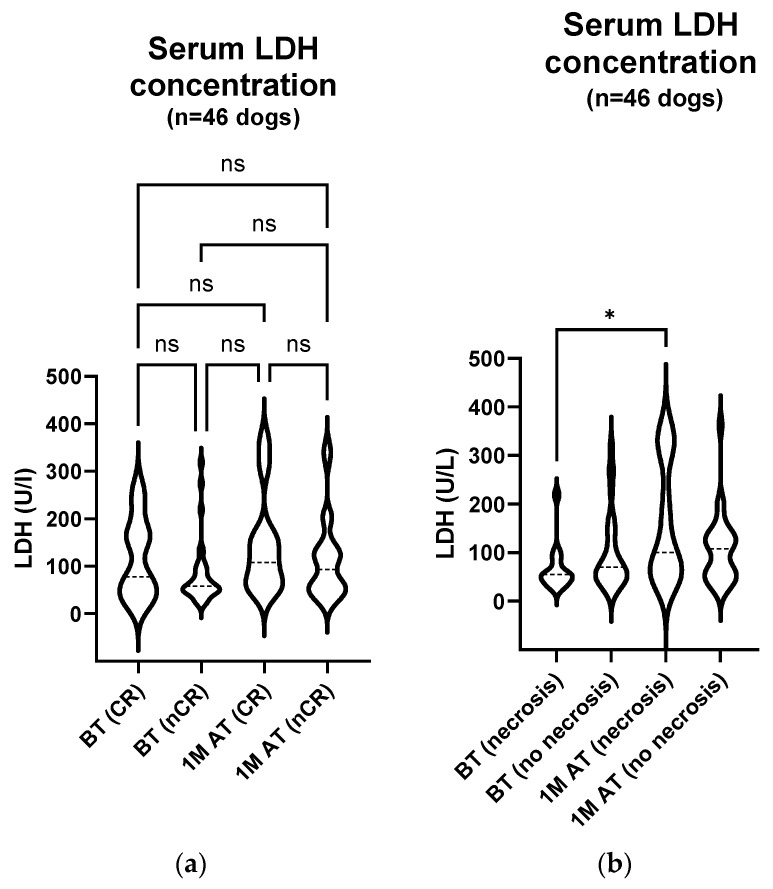
(**a**) **Serum LDH activity comparison: pre-treatment and one month post treatment in correlation with treatment response in 46 dogs.** The enzyme activity was measured with an automated biochemical analyser on the day of treatment and four weeks later. The analysis showed no statistically remarkable changes in the serum LDH activity (*p*: BT (CR) vs. BT (nCR) > 0.999; BT (CR) vs. 1 M AT (CR) > 0.999; BT (CR) vs. 1 M AT (nCR) > 0.999; BT (nCR) vs. 1 M AT (CR) = 0.117; BT (nCR) vs. 1 M AT (nCR) = 0.370; 1 M AT (CR) vs. 1 M AT (nCR) > 0.999). BT, before treatment; 1 M AT, one month after treatment; CR, complete response; nCR, non–complete response (comprising of PR, SD, and PD); ns, not significant. (**b**) **Serum LDH activity comparison: pre-treatment and one month post treatment in correlation with treatment-related enhanced tumour necrosis in 46 dogs.** The enzyme activity was measured with an automated biochemical analyser on the day of treatment and four weeks later. The analyses showed a statistically significant increase in the serum LDH activity one month after treatment (*p*: BT (necrosis) vs. BT (no necrosis) > 0.999; BT (necrosis) vs. 1 M AT (necrosis) = 0.048; BT (necrosis) vs. 1 M AT (no necrosis) = 0.284; BT (no necrosis) vs. 1 M AT (necrosis) = 0.628; BT (no necrosis) vs. 1 M AT (no necrosis) > 0.999; 1 M AT (necrosis) vs. 1 M AT (no necrosis) > 0.999). BT, before treatment; 1 M AT, one month after treatment; * *p* < 0.05.

Also, the serum LDH activity before and one month post treatment was compared between the 11 dogs with CR (median 77.50 U/L (44.00–174.5 U/L) before treatment and median 108.0 U/L (65.00–171.0 U/L) one month after treatment) and the remaining 35 dogs with nCR (either PR, SD or PD) at that time point (median 58.00 U/L (46.00–91.50 U/L) before treatment and median 93.00 U/L (48.50–137.0 U/L) one month after treatment); no statistically significant changes were observed between the respective groups (Figure 7a; *p*: BT (CR) vs. BT (nCR) > 0.999; BT (CR) vs. 1 M AT (CR) > 0.999; BT (CR) vs. 1 M AT (nCR) > 0.999; BT (nCR) vs. 1 M AT (CR) = 0.117; BT (nCR) vs. 1 M AT (nCR) = 0.370; 1 M AT (CR) vs. 1 M AT (nCR) > 0.999).

One month after treatment, the enhanced necrosis of tumour tissue was observed in 14 of the 46 dogs. We compared the serum LDH activity between these 14 animals before and one month after treatment (median 54.75 U/L (39.88–81.00 U/L) before treatment and median 100.3 U/L (56.50–313.8 U/L) one month after treatment) and the remaining 32 animals at the same time points (median 70.25 U/L (46.75–134.3 U/L) before treatment and median 107.8 U/L (48.13–136.5 U/L) one month after treatment). A statistically significant increase in the serum LDH activity was observed in the dogs in which treatment caused the necrosis of tumour tissue (Figure 7b; (*p*: BT (necrosis) vs. BT (no necrosis) > 0.999; BT (necrosis) vs. 1 M AT (necrosis) = 0.048; BT (necrosis) vs. 1 M AT (no necrosis) = 0.284; BT (no necrosis) vs. 1 M AT (necrosis) = 0.628; BT (no necrosis) vs. 1 M AT (no necrosis) > 0.999; 1 M AT (necrosis) vs. 1 M AT (no necrosis) > 0.999).

## 4. Discussion

The efficacy of ECT has been proven not only in the treatment of canine MCT, but also in various other tumour types [58,59,60]. In combination with IL-12 GET, this treatment approach is further improved and leads to better treatment results, as previous studies have shown [1,2,3,4,5,6]. Although the evaluation of its antitumour effectiveness was not the primary aim of this study, we examined the treatment outcomes at both one- and six-months post treatment. The findings of our study are consistent with previously published results and emphasise the treatment efficacy [6]. However, the focus of this study was on investigating possible changes in selected biomarkers and their clinical utility as predictive indicators of treatment success. Therefore, a long-term follow-up was not conducted.

To begin with, one of the possible biomarkers observed in this study was serum ferritin, in part because elevated serum ferritin levels have been observed in dogs with various malignancies, including lymphomas, histiocytic sarcomas, mammary tumours, IMHA and liver disease [26,27,28,29]. The aetiology of hyperferritinaemia is not fully known. It is likely to be complex in both neoplastic and non-neoplastic diseases. The proposed mechanisms include factors such as erythrocyte degradation, alterations in erythropoietic processes, release due to tissue damage and hepatocyte injury. It has already been demonstrated that malignant cells are able to synthesise ferritin [61].

There is a publication describing that mast cells in human skin store ferritin in their secretory granules and release it rapidly when activated by various mediators [62]. However, to the authors’ knowledge, changes in the serum ferritin concentrations in dogs have not been associated with the presence of MCT or with the assessment of the response to treatment using an ablative technique, such as ECT alone or in combination with IL-12 GET.

In our study, we did not detect significant changes in the serum ferritin concentration before and after treatment, regardless of the response to treatment or the presence of enhanced tumour necrosis. This suggests that the serum ferritin concentration may not be a reliable biomarker for assessing the response to ECT and IL-12 GET treatment in dogs with MCT. Nevertheless, we cannot exclude ferritin as a biomarker in dogs with MCT, as we did not compare the serum ferritin levels with a group of healthy dogs.

Furthermore, the nucleosome concentration was also analysed in the treated patients. Plasma nucleosome concentrations have been evaluated as a potential diagnostic or predictive biomarker because elevated nucleosome concentrations have previously been observed in various malignancies in humans and in certain malignancies in dogs, including lymphomas, haemangiosarcomas, histiocytic sarcomas and malignant melanomas, suggesting their utility as a diagnostic biomarker for early detection in dogs [35,36,40,41,42]. However, in a recent study, the serum nucleosome concentration did not prove to be a reliable diagnostic biomarker for MCT, accurately predicting only 19% of MCT cases. Interestingly, the study showed that high-grade MCTs had a significantly higher mean nucleosome concentration than low-grade MCTs and healthy dogs [35]. In our study, we measured the nucleosome concentration in 43 patients with confirmed mast cell neoplasia before treatment and found that the assay used accurately predicted the presence of the disease in 23.3% of patients (high concentrations), while 27.9% were in the grey zone. This study showed that the assay was more sensitive compared to previous reports; however, the number of patients observed in our study was lower. The specificity of the assay could not be evaluated in comparison to previous studies, as there was no healthy control group.

However, we refrained from comparing the nucleosome concentrations between different tumour grades as several tumours in our study were subcutaneous, and therefore such a classification is not possible, or were cutaneous, but no histopathology was performed. Most cutaneous MCTs in our study that were histopathologically graded were classified as grade II (Patnaik) and low grade (Kiupel). It is worth noting that, in this study, accurate grading was less reliable in animals with multiple MCTs, as in these animals only one tumour was sampled for grading. In addition, a previous study has shown that grading based on punch biopsy, as we performed, compared to excisional biopsy has 100% agreement with the Patnaik grading but only 95% agreement with the Kiupel grading [63]. Consequently, there is a possibility that the grade is underestimated when relying on punch biopsy to estimate the grade of the tumour, especially using the Kiupel grading standards.

On the other hand, we did observe a significant increase in Patnaik grading plasma nucleosome concentrations in 43 MCT patients one month after treatment with ECT and IL-12 GET. Looking at the measured values six months after treatment, they were not significantly changed in 17 out of 43 dogs. But at the same time, the values in the same 17 patients one month after treatment were no longer significant. This can be explained by the fact that a much smaller number of animals were included in the latter comparison. This raises the question of whether the change in the serum nucleosome concentration six months after treatment could be statistically significant if more patients were included.

A comparison between the response to treatment one month after treatment and changes in the plasma nucleosome concentrations showed no correlation. On the other hand, the presence of enhanced tumour necrosis at the same time point correlated significantly positively with the plasma nucleosome concentrations. Nucleosomes are predominantly located within cells and are released into the bloodstream, particularly upon activation or cell death [36]. This may explain the increase in the plasma nucleosome concentrations in patients where ablative techniques such as ECT with IL-12 GET were used for treatment, resulting in tissue inflammation and the necrosis of the tumour. In a recent study, Wilson-Robles et al. examined the nucleosome levels in 528 dogs with various common malignancies and compared them to 134 healthy dogs. Although elevated nucleosome levels have been found least frequently in soft tissue sarcomas, osteosarcomas and MCT [35], we could hypothesize that the increase in the plasma nucleosome concentration is due to tumour tissue degradation, regardless of the tumour type. Very similar changes in the nucleosome concentration during treatment with either radiotherapy or chemotherapy have been observed in human medicine in various malignancies [64,65,66]. The fluctuation in the plasma nucleosome concentration should be further investigated in different tumour types in dogs.

Overall, the results suggest that nucleosomes may be useful in assessing early treatment effects, particularly in the context of enhanced tumour necrosis. Their use as reliable biomarkers for predicting the long-term response to treatment needs further investigation. Since nucleosomes are part of the emerging concept of liquid biopsy, measuring circulating tumour DNA, circulating tumour cells and tumour extracellular vesicles in the blood of these animals could also provide further insight into disease progression or the treatment outcome [67].

Further on, the serum LDH activity was measured. Elevated serum LDH levels are associated with various cancers and are considered an indicator of high metabolic activity in malignant tumours [30,31]. In veterinary medicine, LDH has been studied in lymphomas, mammary gland tumours and oral cavity tumours, especially oral malignant melanoma [30,31,32,33,34].

In our study, a significant increase in the serum LDH activity was observed in 46 patients one month after treatment. We also observed changes in the LDH activity six months after treatment, albeit in a smaller group of 18 dogs examined at this time. However, in these 18 dogs, there was no significant change in the serum LDH activity six months after treatment, either compared to the pre-treatment values or compared to the period one month after treatment. Furthermore, a comparison of the LDH activity between the day of treatment and one month after treatment also showed no significant difference in these 18 dogs. As mentioned with regard to the plasma nucleosome concentration, it is important to consider the potential impact of the small sample size on these observations.

The serum LDH activity did not differ significantly between patients who responded to treatment and those who did not, as observed for the plasma nucleosome concentrations. When comparing the presence of enhanced tumour necrosis one month after treatment and the serum LDH activity, a significant increase was observed. In a study by Marconato et al., they found no association between significantly elevated LDH levels and the presence of MCT or MCT metastases in dogs when comparing healthy animals, animals with non-neoplastic disease and dogs with cancer [31]. A similar observation was made in our study, where only 10.8% of our patients with cytologically or histopathologically confirmed MCT had increased serum LDH activity before treatment. Therefore, the increase in LDH activity after one month of treatment and in the presence of enhanced tumour necrosis could be due to tissue breakdown, which is specifically greatest in the period up to one month after treatment with ECT and IL-12 GET. This suggests that the serum LDH activity may reflect short-term treatment effects but may not serve as a reliable long-term predictive biomarker for assessing the treatment response in dogs with MCT treated with ECT and IL-12 GET. Furthermore, the lack of correlation between the increased serum LDH activity and the presence of MCT suggests that the measurement of the serum LDH activity is not a useful diagnostic biomarker for determining the presence of MCT.

Finally, during the observation period, it was often detected that a higher number of PR and SD treatment responses occurred one month after treatment, accompanied by general signs of inflammation in the treated area. However, as healing progressed, the treatment responses generally became satisfactory. Similar observations were also made in a recent study, suggesting that the assessment of the treatment response after one month may be premature for the ECT combined with GET treatment approach, as the immune response is still active at this time, as evidenced by persistent swelling [6]. In light of this consideration, the comparison between the CR and nCR groups one month after treatment is less meaningful, as persistent swelling may interfere with the accurate assessment of treatment outcomes (based on RECIST criteria) and a comparison at a later date may be more meaningful.

### Study Limitations

A major limitation of this study is the lack of samples six months after treatment in patients with disease progression. For a more meaningful comparison, it would be beneficial to have a larger number of samples from patients with stable or progressive disease six months after treatment to observe possible trends or differences in the measured biomarkers between patients with different long-term treatment outcomes.

Limitations also arise from the practise of sampling only one tumour for grading in animals with multiple tumours. This approach may underestimate the grade of the other present tumours, considering that different mast cell tumours may have different grades in the same animal.

A further limitation results from the inconsistent and incomplete clinical staging of the regional lymph nodes and the incomplete tumour grading. Tumour biopsy and histopathological grading were not performed in all patients. When tumours were assessed cytologically, no grading criteria were applied [68]. The surgical removal of regional lymph nodes or their sampling for the cytological determination of the presence of metastases were also not consistently performed, and the cytological grading criteria for lymph nodes were not applied [69]. Therefore, the possible influence of the tumour grade or metastatic disease on the measured biomarkers cannot be definitively excluded.

The lack of a healthy patient group for comparison is another important limitation of this study. Given the fact that markers were not monitored in patients with stable or progressive disease, and the lack of sufficient patient numbers, especially in the groups where trends were observed, further studies in larger patient groups are warranted.

Finally, the comparison of the selected biomarkers before and one month after treatment, based on the response to treatment according to the RECIST criteria, may not be meaningful in this study. The ongoing immune response within the tumour remains active and directly influences the assessment of the response to treatment and therefore represents a limitation of this study.

## 5. Conclusions

In summary, this study investigated the potential of plasma nucleosome and serum ferritin concentrations, as well as serum LDH activity, to be used as biomarkers for predicting the response to treatment of dogs with MCT using a combination of ECT and IL-12 GET. The plasma nucleosome concentration and serum LDH activity were found to be promising early indicators of treatment effects, especially in animals with enhanced tumour necrosis (Figure 1b), but their long-term predictive value needs further investigation. The serum ferritin concentration showed no significant predictive potential for the treatment response in this context.

Overall, this study contributes valuable insights into the use of biomarkers in veterinary oncology and highlights the need for further research to identify reliable predictors of the treatment response in dogs with MCT or other malignancies undergoing ECT and IL-12 GET therapy. The further investigation and validation of potential predictive biomarkers, as well as research into additional markers, could ultimately improve decision making in the treatment of canine cancer patients and improve treatment outcomes.

## Figures and Tables

**Table 2:** List of patients in whom the nucleosome and ferritin concentrations and LDH activity were determined.

Patient Number	Nucleosomes		Ferritin		LDH		Patient Number	Nucleosomes		Ferritin		LDH	
	0 and 1 M	6 M	0 and 1 M	6 M	0 and 1 M	6 M		0 and 1 M	6 M	0 and 1 M	6 M	0 and 1 M	6 M
1	✓	✓	✓		✓	✓	25	✓	✓	✓	✓	✓	✓
2	✓		✓		✓		26	✓		✓		✓	
3	✓		✓		✓		27			✓		✓	✓
4	✓	✓	✓	✓	✓	✓	28	✓	✓	✓		✓	✓
5	✓		✓		✓		29	✓	✓	✓		✓	✓
6	✓		✓		✓		30	✓		✓		✓	
7	✓	✓	✓	✓	✓	✓	31	✓				✓	✓
8	✓		✓		✓		32	✓				✓	
9	✓	✓	✓	✓	✓	✓	33	✓				✓	✓
10	✓	✓	✓	✓	✓	✓	34	✓				✓	
11	✓	✓	✓	✓	✓	✓	35	✓		✓		✓	
12	✓	✓	✓	✓	✓	✓	36	✓		✓		✓	
13	✓	✓	✓	✓			37	✓		✓		✓	
14	✓	✓	✓	✓	✓	✓	38	✓		✓		✓	
15	✓	✓	✓		✓	✓	39	✓				✓	
16	✓	✓	✓		✓		40	✓				✓	
17	✓				✓		41	✓				✓	
18	✓				✓		42	✓					
19	✓	✓			✓	✓	43	✓				✓	
20	✓	✓			✓	✓	44	✓				✓	
21	✓		✓		✓		45					✓	
22	✓		✓		✓		46					✓	
23	✓		✓	✓	✓		47					✓	
24	✓	✓	✓		✓	✓	48					✓	

LDH, lactate dehydrogenase; 0 and 1 M, the day of treatment and one month after treatment; 6 M, six months after treatment; ✓, blood sampling and determination of potential biomarker.

**Table 3:** Tumour response one- and six-months post treatment and overall response one- and six-months post treatment.

Patient Number	Site of the Tumours	Tumour Response One Month Post Treatment	Tumour Response Six Months Post Treatment	Overall Response One Month Post Treatment	Overall Response Six Months Post Treatment	Necrosis Observed at One Month Post Treatment
1	Left hind leg	SD	PR	SD	PR	✓
2	Left front leg	PR	PD	PR	PD	✓
3	Left front leg	PR	PD	PR	PD	✓
4	Right inguinal region	CR	CR	CR	CR	✓
5	Right hind leg	PR	CR	PR	SD	
	Right scapula	PR	CR			
	Left hind leg	PR	RT			
	Left inguinal region	PR	CR			
6	Right hind leg	SD	RT	SD	RT	
7	Left scapula	CR	CR	CR	CR	
8	Right dorsum	PR	RT	PR	RT	
9	Left hind leg	PR	CR	PR	CR	
10	Right front leg	CR	CR	CR	CR	✓
11	Left ear	CR	CR	CR	CR	
	Dorsal part of the neck	CR	CR			
	Right abdominal region	CR	CR			
	Perianal region	CR	CR			
12	Prepuce	CR	CR	CR	CR	
13	Left front leg	CR	CR	CR	CR	✓
	Right lip commissure	CR	CR			
14	Dorsum	CR	CR	CR	CR	
	Left abdominal region	CR	CR			
	Left hind leg	CR	CR			
	Left hind leg	CR	CR			
	Tail base	CR	CR			
	Vulva	CR	CR			
15	Left elbow	CR	CR	CR	CR	
16	Right inguinal region	PR	CR	PR	CR	✓
17	Chin	SD	RT	SD	RT	
	Right thoracic region	CR	CR			
	Right hind leg	SD	RT			
	Right hind leg	CR	CR			
18	Perianal region	CR	PD	CR	PD	
19	Left hind leg	PR	CR	PR	CR	
20	Left abdominal region	PR	PR	PR	PR	
	Left front leg	PR	PR			
	Right hind leg	PR	PR			
21	Right hind leg	CR	CR	CR	CR	
22	Right orbital region	PR	LTFU	PR	LTFU	
	Perianal region	CR	LTFU			
	Perianal region	CR	LTFU			
	Right hind leg	CR	LTFU			
23	Right front leg	PR	PR	SD	PD	
	Left front leg	PR	CR			
	Sternal region	SD	SD			
	Right orbital region	PR	CR			
24	Right hind leg	PR	CR	PR	CR	
25	Prepuce	CR	CR	PR	CR	
	Right hind leg	CR	CR			
	Perianal region	CR	CR			
	Right abdominal region	CR	CR			
26	Prepuce	SD	PD	SD	PD	
27	Right ear	PR	CR	PR	CR	
28	Left ear	PR	CR	PR	CR	
29	Left abdominal region	PR	CR	PR	CR	
30	Left thoracic region	CR	CR	CR	RT	
	Left thoracic region	CR	CR			
	Left hind leg	CR	CR			
	Right ear	CR	CR			
	Right thoracic region	CR	CR			
	Left inguinal region	CR	CR			
31	Left hind leg	PR	CR	PR	CR	
32	Tail	CR	CR	CR	CR	
33	Right front leg	PR	CR	PR	CR	✓
34	Left hind leg	SD	RT	SD	RT	
	Left hind leg	SD	RT			
	Scrotum	SD	RT			
	Prepuce	SD	RT			
	Left front leg	SD	RT			
35	Prepuce	PR	CR	SD	RT	
	Right hind leg	PR	CR			
36	Right front leg	PR	CR	PR	CR	✓
37	Right abdominal region	PR	PR	PR	PR	✓
	Perianal region	PR	PR			
38	Right lip corner	PR	CR	PR	CR	
39	Left front leg	PR	LTFU	PR	LTFU	✓
	Left front leg	PR	LTFU			
40	Frontal region	CR	CR	CR	CR	✓
41	Left ear	SD	RT	SD	RT	
42	Left hind leg	SD	SD	SD	SD	
43	Perianal region	PR	PR	PR	PR	
44	Right hind leg	PR	CR	PR	CR	
45	Left axillary region	PR	CR	PR	CR	✓
46	Right hind leg	PR	CR	PR	CR	✓
47	Right lip commissure	CR	CR	CR	CR	
48	Right hind leg	SD	PD	SD	PD	

CR, complete response; PR, partial response; SD, stable disease; PD, progressive disease; RT, re-treatment prior to response evaluation; LTFU, lost to follow-up; ✓, necrosis observed. Evaluation of response to treatment is based on the RECIST criteria [21].

## Data Availability

The data presented in this study are available on request from the corresponding author.
